# Threat Upon Entry: Effect of Coworker Ostracism on Newcomers’ Proactive Behaviors During Organizational Socialization

**DOI:** 10.3389/fpsyg.2021.545478

**Published:** 2021-04-06

**Authors:** Pan Liu, Yihua Zhang, Yan Ji, Shaoxue Wu

**Affiliations:** ^1^School of Economics and Management, Beijing Jiaotong University, Beijing, China; ^2^Graduate School, Pepperdine University, Los Angeles, Los Angeles, CA, United States; ^3^CCCC Wuhan Harbour Engineering Design & Research Corporation Limited, Wuhan, China; ^4^School of Management, Universiti Sains Malaysia, Pulau Pinang, Malaysia

**Keywords:** coworker ostracism, organizational socialization, newcomers’ proactivity, psychological availability, emotional intelligence

## Abstract

Extant literature has underlined the importance of newcomer proactive socialization to the organization. However, the effect of coworker ostracism on newcomers’ proactive behaviors has not been noticed. Drawing on the conservation of resources (COR) theory, we proposed a model exploring how coworker ostracism impacted newcomers’ proactive behaviors via the mediation of psychological availability. Through an empirical study with a sample of 263 newcomers and three waves of longitudinal data, we found that coworker ostracism had a negative effect on newcomers’ information seeking and *guanxi* developing. In addition, emotional intelligence enhanced the negative effect of coworker ostracism on newcomers’ psychological availability and the indirect influence of coworker ostracism on newcomers’ proactive behaviors via psychological availability. Important theoretical and practical implications are discussed.

## Introduction

Ostracism, defined as the extent to which individuals perceive that they are excluded by others (Williams, 1997, 2001; Williams and Nida, 2011), is a common phenomenon in the workplace. A survey of 262 employees illustrated that 66% of respondents experienced exclusion over a 5-year period (Fox and Stallworth, 2005). Evidence has also illustrated that ostracism can result in physically colder (Williams and Nida, 2011), psychological discomfort (Williams and Jarvis, 2006), and sadness (Howard et al., 2019). Given its power, significant research effort has been devoted to further understanding the nature and impacts of ostracism (Baumeister et al., 2005; Hitlan et al., 2006; Hitlan, 2009; Leung et al., 2011; Wu et al., 2012; Robinson et al., 2013; Gerber and Wheeler, 2014; Peng and Zeng, 2017; Quade et al., 2017; Yang and Treadway, 2018).

Despite these progresses in ostracism study, previous approaches to examining ostracism can still be extended in several ways. First, the effect of ostracism on newcomers’ proactive socialization has rarely been examined in research and management practice. Although some scholars have illustrated the influence of ostracism on employees’ attitudes and behaviors (Leung et al., 2011; Wu et al., 2012; Balliet and Ferris, 2013; Zhu et al., 2017), its role in organizational socialization has not been revealed. Moreover, previous research has indicated that interaction with insiders is beneficial for newcomers’ socialization (Morrison, 2002; Kammeyer-Mueller and Wanberg, 2003; Jokisaari and Nurmi, 2009). However, less research has focused on the role of coworkers in socialization than that of supervisors. Actually, coworkers are an important source of social influence and can directly or indirectly affect newcomers (Chen et al., 2013). Hence, it is significant to explore the effect of coworker ostracism toward newcomers in socialization.

To fill in these gaps, we selected psychological availability that refers to individuals’ perception of the physical, emotional, or psychological resources to engage at work (Kahn, 1990; May et al., 2004) as the transformation mechanism of how coworker ostracism impacts newcomers’ proactive socialization based on conservation of resources (COR) theory (Hobfoll, 1989). The COR theory suggests that negative work-related experiences that result in psychological strain can cause depletion of resource (Hobfoll, 1989, 2001). Individuals usually avoid further resource expenditure to protect their remaining resources when facing the shortage of resources. According to this theory, we argue that coworker ostracism may deplete the resources of newcomers, prohibiting their psychological availability, which in turn impede their proactive behaviors. Moreover, the impact of coworker ostracism on newcomers may be different among newcomers due to emotional intelligence (Carmeli et al., 2009). Emotionally intelligent newcomers are sensitive to others’ emotions, and can regulate themselves to return quickly to normal psychological states (Law et al., 2004). However, we deem that regulating emotions may deplete the resources and further impact subsequent self-control tasks. Hence, newcomers’ emotional intelligence is operationalized as a moderator to explore relationship between coworker ostracism toward newcomers and their proactive organizational behaviors.

In our study, we try to make at least three contributions to the extant literature. First, we contribute to the research on socialization by illustrating the negative impact of ostracism on newcomers. Organizational entry is a high-pressure situation for newcomers and such negative experiences can prohibit newcomers from successful adjustment (Ashforth et al., 2007). In this regard, turnover actually occurs more among newcomers. Because ostracism has negative and detrimental effects on newcomers, comprehending ostracism in the newcomer context is of great significance. Second, our study advances the ostracism literature by illustrating whether, how and under what conditions coworker ostracism impacts newcomers’ proactive socialization. In doing so, our study enriches ostracism literature by exploring its downstream effects on socialization. Third, this study extends the COR theory by revealing the mediating role of psychological availability. From the perspective of psychological resources, this study provides a new theoretical perspective and explanation for how coworker ostracism impacts newcomers’ proactive socialization via psychological availability.

## Theory and Hypotheses

### Conservation of Resources Theory

Conservation of resources theory is introduced as a theoretical framework for understanding and explaining the causes and consequences of psychological stress (Hobfoll and Shirom, 2001). According to COR theory, resources are defined as objective resources, conditions, personal characteristics, or energy sources that are beneficial to achieve individual’s tasks or goals (Hobfoll, 1989). The basic tenet of COR theory is “individuals are motivated to retain, protect, and foster what they value” (Hobfoll, 2001; Westman et al., 2004). When confronted with stress, individuals usually strive to minimize net loss of resources and protect against further loss (Hobfoll, 1989). Scholars have posited that withstanding stress or impulses requires self-control resource investment and then depletes individuals’ resource (Marcus and Schuler, 2004; Stucke and Baumeister, 2006; Christian and Ellis, 2011).

**FIGURE 1:**
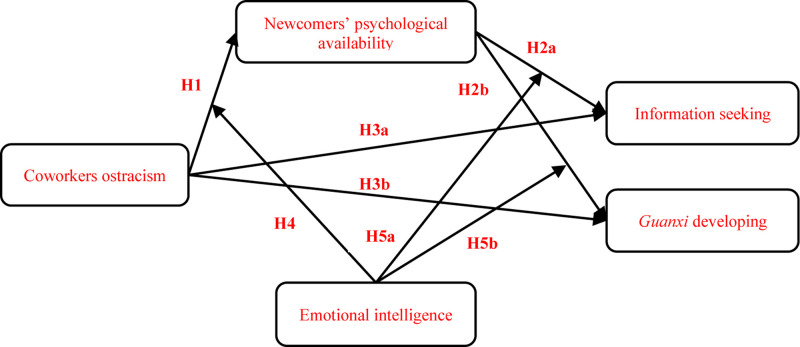
Conceptual model.

Ostracism can be interpreted as a workplace stressor that induces deleterious strain and behavioral problems among employees (Twenge et al., 2003). Newcomers may feel anxiety and uneasy when excluded by coworkers. However, perceptions of the risks involved prevent them from retaliating in response to the coworkers’ misbehaviors (Wang and Kim, 2013). Drawing on the COR theory, newcomers in this situation have to save psychological resources to continuously control their emotions and avoid saying anything inappropriate. Consequently, newcomers have not sufficient resources to seeking information or developing *guanxi*. Thus, COR theory is particularly relevant to this study because it provides a comprehensive framework to understand why newcomers fail to take initiatives after excluded by coworkers. The conceptual model is shown in Figure 1.

### Coworker Ostracism and Psychological Availability

As a uniquely painful experience (Robinson et al., 2013), ostracism has negative effects on employees’ behaviors and attitudes. Some studies have shown that ostracism is negatively related to psychological well-being (Xu et al., 2015; Zhang and Shi, 2017) and belongingness (O’Reilly and Robinson, 2009; O’Reilly et al., 2015), but positively related to emotional exhaustion (Wu et al., 2012), job tension (Zhu et al., 2017), and depression (Ferris et al., 2008; Scott and Duffy, 2015). Ostracized employees may enter a state of distress, anxiety, depression, and possibly even meaninglessness (Wu et al., 2012; O’Reilly et al., 2015). In the organization, coworkers can affect the newcomers’ engagement in aggressive behavior, retaliation, undermining, and organizational citizenship behavior (Chen et al., 2013). In this case, we deem that coworker ostracism can have direct effects on newcomers’ psychological availability.

Psychological availability is a reflection of individuals’ physical, emotional, or psychological resource level (May et al., 2004). Previous studies have noted that positive social interaction in the workplace can exert positive effects on employees’ psychological state (i.e., psychological availability) and generate desirable outcomes (Collins, 2004; Heaphy and Dutton, 2008), while coping with perceived uncertainty and stress can inhibit psychological availability. According to COR theory, stress is a critical factor that results in resource loss (Oaten and Cheng, 2005). Coworker ostracism can cause extreme stress on newcomers and make them fall into anxiety (Balliet and Ferris, 2013). In this situation, newcomers have to consume limited resources to deal with stress, and in turn, psychological availability plunges. In addition, coworker ostracism can bring negative emotions to newcomers. Excluded newcomers feel isolated and neglected, which can damage their self-esteem and self-confidence (Thau and Mitchell, 2010). Controlling negative emotions and focusing on work tasks can consume resources and decrease the psychological availability. Thus, we propose:

Hypothesis 1: Coworker ostracism is negatively related to psychological availability of newcomers.

### The Mediating Role of Psychological Availability

Psychological availability reflects a state in which individuals are able to direct psychological, intellectual, and emotional resources into job performance (Kahn, 1990), and it can help individuals tackle the extra requirements necessary for proactive behaviors. When individuals are psychologically available, they have physical, emotional, or psychological resources and thus increased energy to take initiatives. Consequently, we assumed that psychological availability is positively related to newcomers’ proactive socialization. Newcomer proactivity is the means by which newcomers actively affect their work environment through initiative actions such as seeking information about their role and work environment to reduce uncertainty (Ashforth et al., 2007). In addition, we also consider *guanxi* developing as a type of proactive socialization because it has been widely emphasized in Chinese context by researchers (Wang and Kim, 2013).

Information seeking referring that newcomers seek for and acquire information about the work and organization (Morrison, 1993a,b; Ashford and Black, 1996) has received the most attention in previous research on newcomer proactivity. Newcomers need information about formal and informal rules and norms of the organization, so they can achieve their performance goals and adjust into the organization (Kowsikka and James, 2019). Information seeking requires cognitive resources to identify information source, psychological resources to cope with failures and overcome resistance in others, and proactive engagement in seeking to acquire important information. Only newcomers who psychologically availably usually have sufficient resources and capable of regulating their behaviors (Kahn, 1990), which helps them focus on searching for information. Thus, we propose:

Hypothesis 2a: Psychological availability is positively related to newcomers’ information seeking.

*Guanxi* developing is defined as the establishment of an informal interpersonal relationship characterized by an unlimited exchange of favors between two individuals (Tsui and Farh, 1997; Wang and Kim, 2013; Chang, 2014). In consistence with relationship building, *guanxi* developing enables newcomers to interact frequently with insiders, which increases newcomers’ role identification and expectations (Farh et al., 1998; Wang and Kim, 2013). Thus, *guanxi* developing can be a crucial task for most Chinese newcomers (Chen and Tjosvold, 2007). Nevertheless, *guanxi* developing consumes resources. Psychological availability provides the vital resources for newcomers to developing *guanxi*. Newcomers with psychological availability make better relationship partners because they are better able to adapt to their partners and form social bonds (Finkel and Campbell, 2001). In contrast, those without psychological availability may perform less well in social interactions and are more likely to have conflicts with insiders (Palumbo et al., 1992; Denson et al., 2011). Thus, we propose:

Hypothesis 2b: Psychological availability is positively related to newcomers’ guanxi developing.

According to the COR theory, individuals who suffer from a lack of resources can take a defensive posture to conserve their remaining resources and avoid further losses (Hobfoll and Freedy, 1993; Halbesleben and Bowler, 2007; Halbesleben et al., 2014). In this instance, individuals cannot be motivated to maximize their performance in other areas. When newcomers are exposed to coworker ostracism, they require to invest resources to cope with that. Naturally, this process results resource loss and then decreases psychological availability. Decline in psychological availability prohibits newcomers from seeking information and developing guanxi. Thus, we propose:

Hypothesis 3a: Psychological availability mediates the negative influence of coworker ostracism on newcomers’ information seeking.

Hypothesis 3b: Psychological availability mediates the negative influence of coworker ostracism on newcomers’ guanxi developing.

### The Moderating Role of Emotional Intelligence

Emotional intelligence is defined as the ability to perceive, regulate, and manage emotions so as to promote emotional and intellectual growth (Salovey and Mayer, 1990; Davies et al., 1998; Grover and Furnham, 2020). Emotionally intelligent individuals are well in perceiving and managing others’ emotions. Previous research has indicated that emotional intelligence is related to psychological well-being (Carmeli et al., 2009), task performance (Joseph and Newman, 2010), positive moods, and higher self-esteem (Schutte et al., 2002). Nevertheless, it is not sure whether emotionally intelligent newcomers can maintain themselves when confronted with coworker ostracism.

First, negative emotions and stress can deplete resources, and make impulsive behavior more likely. According to COR theory, suppressing negative emotions or aggressive impulses is an effortful process that requires to invest psychological and cognitive resource (Marcus and Schuler, 2004). Newcomers who are in high emotional intelligence are very sensitive to others’ emotions, so they may sharply perceive the negative emotion or aggression behind coworker ostracism, which makes them feel anxious and stressed (Law et al., 2004). Thus, emotionally intelligent newcomers are more likely to invest resources to cope with that stress when confronted with exclusion from coworkers.

Additionally, emotionally intelligent newcomers are able to recover from emotional distress rapidly (Salovey and Mayer, 1990). However, regulating emotion apparently depletes the psychological resource preparing for other tasks (Halbesleben et al., 2014). Confronted with coworker ostracism, emotionally intelligent newcomers can timely regulate their emotions and avoid losing temper by consuming the resource, but the recovery of resource is not instant and takes a period of time. In other words, such an excluded newcomer is less likely to be psychologically available. Hence, we propose:

Hypothesis 4: Emotional intelligence moderates the relationship between coworker ostracism and newcomers’ psychological availability. When newcomers’ emotional intelligence is higher, the negative effect of coworker on newcomers’ psychological availability is stronger.

The above arguments represent an integrated framework in which psychological availability mediates the negative relationship between coworker ostracism and newcomers’ proactive behaviors (i.e., information seeking and *guanxi* developing), and emotional intelligence moderates the relationship between coworker ostracism and psychological availability. It is logical to believe that emotional intelligence also moderates the strength of the mediator function of psychological availability for the relationship between coworker ostracism and newcomers’ proactive behaviors. As we predict a stronger relationship between coworker ostracism and psychological availability among newcomers with higher emotional intelligence, the negative indirect effect of coworker ostracism on newcomers’ proactivity via psychological availability should be stronger among emotionally intelligent newcomers. That is, emotionally intelligent newcomers are more likely to deplete resources to recover from coworker ostracism, which in turn has a negative effect on newcomers’ proactive socialization. Hence, we propose:

Hypothesis 5a: Emotional intelligence enhances the indirect effect of coworker ostracism on newcomers’ information seeking via psychological availability.

Hypothesis 5b: Emotional intelligence enhances the indirect effect of coworker ostracism on newcomers’ guanxi developing via psychological availability.

## Materials and Methods

### Sample and Procedure

We tested our conceptual model with a sample from a large financial company in China. According to previous research (Bauer and Green, 1994; Allen, 2006; Bauer et al., 2007; Ou et al., 2018), newcomers are defined as employees who have worked for their organizations for less than 1 year. Through interviews with newcomers and coworkers, and discussions with company managers, we found that ostracism was universal in this company. Given this situation, we believed that this company was suitable for our study and appropriate to collect data. After acquiring the permission of managers, we described the survey for respondents on site. With a list of names from HR, codes were assigned to each newcomer. We explained to all participants and guaranteed that the survey was voluntary, confidential, anonymous, and irrelevant to their performance evaluation. Enough time was given to complete the questionnaires. In addition, to reduce social desirability, we reminded the participants of the importance of answering honestly for the sake of our academic research. After completing the questionnaires, the participants sealed them in envelopes and submitted them directly to the research team. To motivate them for their participation, respondents who completed the surveys were given 20 Chinese yuan each time.

Previous research has proved that 2-week separation allows for forming and developing perceptions of the variables (Fulmer and Ostroff, 2017; Liu et al., 2017; Yam et al., 2017; Kim et al., 2018). Thus, we used a three-wave method for the data collection with each wave separated by 2 weeks to minimize potential common method biases and reduce participants’ fatigue (Podsakoff et al., 2003). In Time 1, we distributed 378 questionnaires to newcomers and asked them to report demographics, emotional intelligence, and coworker ostracism. In Time 2, 324 newcomers who responded in Time 1 were asked to report their psychological availability. In Time 3, 287 newcomers who responded in the first two rounds were asked to report information seeking and *guanxi* developing.

The final sample comprised 263 valid questionnaires, with an overall response rate of 69.58%. To examine whether participants’ response versus non-response created any detectable differences in our sample, a multivariate analysis of variance was conducted (Lance et al., 2000). Results indicated that participants in the initial randomly selected sample and in the final sample for hypothesis testing did not differ significantly with regard to age (*t* = 0.353, *p* = 0.724), education (*t* = 0.285, *p* = 0.776), tenure (*t* = 0.412, *p* = 0.681), or gender (χ^2^ = 0.247, *p* = 0.805). Of the 263 participants, 86 (32.7%) were women and 177 (67.3%) were men. There were nine (3.4%) who held a doctoral degree, 164 (62.3%) who were postgraduates, 88 (33.5%) who were undergraduates, and two (0.8%) who had graduated from junior college. The average age was 25.08 years (*SD* = 2.236), and 96.96% of the newcomers had been in paid employment for less than 1 year, with the remaining 3.04% either having had some internship experience or having been in paid employment for a period before going to graduate school.

### Measures

To ensure the validity and appropriateness of the measures in the Chinese context, a standard translation and back-translation procedure was applied to guarantee the equivalence of meaning (Brislin, 1986). For all measures, we used a five-point Likert-type scale ranging from 1 (completely disagree) to 5 (completely agree).

#### Coworker Ostracism

We used a 10-item scale (Cronbach’s α = 0.873) developed by Ferris et al. (2008) to measure coworker ostracism. This scale includes the following sample item: “Please indicated the extent that coworkers avoided you at work.”

#### Psychological Availability

We adopted a seven-item scale (Cronbach’s α = 0.935) developed by Byrne et al. (2016) to assess newcomers’ psychological availability. Sample items include: “I have the emotional resources to personally invest myself into my work role” and “I am free mentally to concentrate on my job.”

#### Emotional Intelligence

We used a 16-item scale (Cronbach’s α = 0.968) developed by Law et al. (2004) to assess emotional intelligence. Sample items include: “I have good understanding of my own emotions” and “I am a good observer of others’ emotions.”

#### Information Seeking

We used a four-item scale (Cronbach’s α = 0.933) developed by Ashford and Black (1996). Sample item includes: “I tried to learn the important policies and procedures in the organization.”

#### *Guanxi* Developing

We adopted a seven-item scale (Cronbach’s α = 0.891) developed by Wang and Kim (2013). Sample item includes: “I maintained an intimate relationship with colleagues who may help myself in the future.”

#### Control Variables

We controlled for an assortment of variables, including age, gender, education, and tenure.

### Analytic Strategy

First, confirmatory factor analyses (CFA) were conducted using Mplus 8.0 (Muthén and Muthén, 2012) to examine the validity of the measures (Hooper et al., 2008). Second, we conducted linear regression analyses and bootstrapping approach (Baron and Kenny, 1986; Preacher and Hayes, 2008) to test for the direct and indirect effect of coworker ostracism in SPSS 25.0 (Hayes, 2013). Finally, we examined the hypothesized moderated mediation model by incorporating emotional intelligence into the model and calculated the conditional indirect effects with bias-corrected confidence intervals (Edwards and Lambert, 2007).

## Results

### Confirmatory Factor Analysis

Confirmatory factor analysis was conducted with Mplus 8.0. As shown in Table 1, all factor loadings exceeded 0.6 and were significant, suggesting that the item validity of measures was acceptable. The composite reliability (CR) of each construct was larger than 0.7, which suggested that CR was acceptable. And the average variance extracted (AVE) by each construct is larger than 0.5, which illustrated that convergence validity was acceptable. The discriminate validity value (square root of AVE) of each construct was larger than Pearson correlation value. Accordingly, all measures appear to exhibit acceptable values and validity.

**TABLE 1:** Results of confirmatory factor analysis of each measure.

Variable	Estimate	CR	AVE	1	2	3	4	5
Coworker ostracism	0.689–0.790	0.877	0.544	***0.738***				
Psychological availability	0.747–0.873	0.936	0.675	−0.619	***0.822***			
Emotional intelligence	0.73–0.845	0.967	0.661	−0.542	0.708	***0.813***		
Information seeking	0.841–0.943	0.934	0.779	−0.388	0.490	0.547	***0.883***	
*Guanxi* developing	0.646–0.794	0.890	0.539	−0.349	0.404	0.441	0.714	***0.734***

**Note:* CR, composite reliability; AVE, average variance extracted. Discriminate validity value of each construct is shown along the diagonal in bold italics.*

### Descriptive Analyses

Means, standard deviations, reliabilities, and zero-order correlations of variables are shown in Table 2. Coworker ostracism toward newcomers is negatively related to psychological availability (*r* = −0.619, *p* < 0.01), information seeking (*r* = −0.388, *p* < 0.01), and *guanxi* developing (*r* = −0.349, *p* < 0.01). Moreover, psychological availability is positively related to information seeking (*r* = 0.490, *p* < 0.01) and *guanxi* developing (*r* = 0.404, *p* < 0.01).

**TABLE 2:** Means, standard deviations, reliabilities, and correlations.

Variable	1	2	3	4	5	6	7	8	9
Age	1								
Gender	0.233**	1							
Education	0.874**	0.190**	1						
Tenure	0.119	–0.017	0.051	1					
Coworker ostracism	0.122*	0.033	0.140*	–0.098	***0.873***				
Psychological availability	–0.070	0.005	–0.090	0.040	−0.619**	***0.935***			
Emotional intelligence	−0.151*	0.031	−0.143*	–0.061	−0.542**	0.708**	***0.968***		
Information seeking	–0.071	0.071	–0.050	0.015	−0.388**	0.490**	0.547**	***0.933***	
*Guanxi* developing	–0.064	0.077	–0.055	0.034	−0.349**	0.404**	0.441**	0.714**	***0.891***
Mean	25.084	0.673	5.684	0.108	2.333	3.706	3.793	3.914	3.656
*SD*	2.236	0.470	0.548	0.285	0.642	0.864	0.722	0.705	0.667

*Note: N* = 263. Gender was coded as 0 = male and 1 = female. Education was coded as 1 = high school or below, 2 = junior college, 3 = undergraduate, 4 = postgraduate, and 5 = Ph.D. Cronbach’s α values are shown along the diagonal in bold italics.

*p < 0.05.

**p < 0.01.

### Test of Hypotheses

Linear regression analysis in SPSS is utilized to test hypotheses 1, 2a, and 2b. As summarized in Table 3, the negative effect of coworker ostracism toward newcomers on psychological availability was significant after including the controls (β = −0.621, *p* < 0.001, model 2). In addition, the significant positive effects of psychological availability on information seeking (β = 0.488, *p* < 0.001, model 4) and *guanxi* developing (β = 0.400, *p* < 0.001, model 6) were revealed. Thus, H1, 2a, and 2b were supported.

**TABLE 3:** Results of multiple regression analysis.

Variable	Psychological		Information		*Guanxi*	
	availability		seeking		developing	
			
	Model 1	Model 2	Model 3	Model 4	Model 5	Model 6
Intercept	0.915	–0.036	0.307	0.459	0.338	0.504
Age	0.007	0.015	–0.063	–0.071	–0.046	–0.053
Gender	0.049	0.052	0.203	0.178	0.213	0.192
Education	–0.201	–0.065	0.201	0.213	0.116	0.120
Tenure	0.155	–0.080	–0.037	0.035	0.038	0.107
Coworker ostracism		−0.621***	−0.391***		−0.347***	
Psychological availability				0.488***		0.400***
*R*^2^	0.011	0.385***	0.163***	0.251***	0.132***	0.174***
Δ*R*^2^	0.011	0.374***	0.148***	0.236***	0.117***	0.158***
*F*	0.696	32.144***	10.007***	17.212***	7.831***	10.803***

*Note:* ***p < 0.001.

**N* = 263.*

All remaining hypotheses were tested using the PROCESS macro in SPSS 25.0 (Hayes, 2013) with a 5000-resample bootstrap method (Preacher et al., 2007). To test hypotheses 3a and 3b, PROCESS model 4 was executed. As shown in Table 4, **5000** resampling bootstrapping revealed significant indirect effect of psychological availability on the “coworker ostracism toward newcomers—information seeking” relationship (*E.S.* = −0.275, *SE* = 0.072, 95% bias-corrected *CI* = [−0.431, −0.150]) as well as “coworker ostracism toward newcomers—*guanxi* developing” relationship (*E.S.* = −0.196, *SE* = 0.062, 95% bias-corrected *CI* = [−0.332, −0.093]). Thus, H3a and H3b were supported.

**TABLE 4:** Psychological availability as mediator in the relationship between coworker ostracism and newcomers’ proactive behaviors.

Variable	Effect		Boot SE	Boot LL95%CI	Boot UL 95% CI
Information seeking	Direct effect	−0.155	0.119	−0.389	0.080
	Indirect effect	−0.275	0.072	−0.431	−0.150
Guanxi developing	Direct effect	−0.165	0.095	−0.351	0.021
	Indirect effect	−0.196	0.062	−0.332	−0.093

**Notes:* all coefficients are unstandardized. SE, standard error; LL, lower level; UL, upper level; CL, confidence interval.*

**FIGURE 2:**
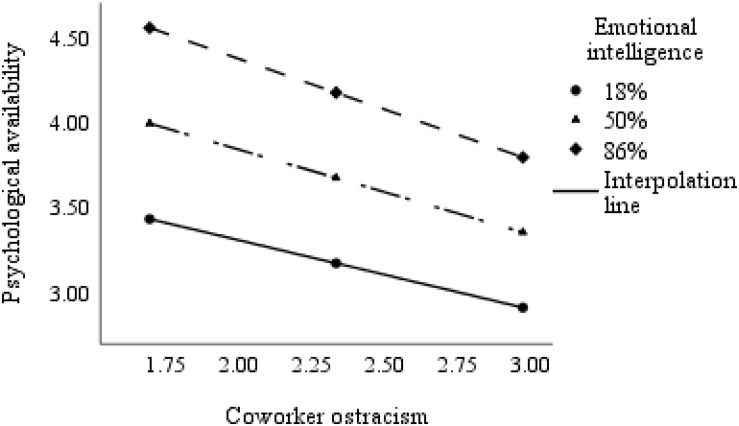
Interactive effect of coworker ostracism and emotional intelligence on newcomers’ psychological availability.

PROCESS model 1 was executed to test H4. Specifically, in PROCESS model 1, one moderator (M) moderates the relationship between the independent variable (X) and dependent variable (Y). As shown in Table 5, it was revealed that the interaction between coworker ostracism toward newcomers and emotional intelligence was significantly related to newcomers’ psychological availability (*E.S.* = −0.130, *SE* = 0.059, 95% bias-corrected *CI* = [−0.246, −0.014]). Following Hayes (2013), we plotted the interactions at 18, 50, and 86% percentiles of emotional intelligence. As shown in Figure 2, the effect of coworker ostracism on psychological availability is stronger for emotionally intelligent newcomer. Thus, H4 was supported.

**TABLE 5:** Emotional intelligence as a moderator in the relationship between coworker ostracism and psychological availability of newcomers.

Variable	Effect	SE	Boot LL 95% CI	Boot UL 95% CI
Y: Psychological availability				
Constant	3.161	0.417	2.340	3.983
M: Emotional intelligence	0.693	0.060	0.575	0.811
X: Coworker ostracism	–0.498	0.091	–0.677	–0.319
Interaction: X × M	–0.130	0.059	–0.246	–0.014

PROCESS model 58 was executed to test hypotheses 5a and 5b. As shown in Table 6, the significant indirect effect of coworker ostracism on information seeking via psychological availability was significant when emotional intelligence was high (*E.S.* = −0.128, *SE* = 0.059, 95% bias-corrected *CI* = [−0.256, −0.030]) but not significant when it was low (*E.S.* = −0.005, *SE* = 0.048, 95% bias-corrected *CI* = [−0.113, 0.079]). And, the indirect effect of coworker ostracism on *guanxi* developing via psychological availability was significant when emotional intelligence was high (*E.S.* = −0.108, *SE* = 0.056, 95% bias-corrected *CI* = [−0.229, −0.016]) but not significant when it was low (*E.S.* = 0.015, *SE* = 0.044, 95% bias-corrected *CI* = [−0.085, 0.093]). Thus, H5a and H5b were supported.

**TABLE 6:** Results of the moderated path analysis.

Dependent	Emotional	Coworker ostracism →			
variables	intelligence	Psychological availability →			
		Dependent variables			
		
		Effect	Boot SE	Boot LL	Boot UL
				95% CI	95% CI
Information	Low	−0.005	0.048	−0.113	0.079
seeking	High	−0.128	0.059	−0.256	−0.030
*Guanxi*	Low	0.015	0.044	−0.085	0.093
developing	High	−0.108	0.056	−0.229	−0.016

## Discussion

Drawing on the COR theory, this study proposed and examined a moderated mediation model to understand the mechanisms through which coworker ostracism toward newcomers impacts their proactive behaviors. Through a multi-wave research design, this study revealed that coworker ostracism could damage newcomers’ proactive behaviors through psychological availability, and emotional intelligence moderated the effect of coworker ostracism on newcomers’ psychological availability. In addition, the empirical results illustrated that emotional intelligence could enhance the indirect effect of coworker ostracism on newcomers’ proactive behaviors.

### Theoretical Implications

In examining these hypotheses, the findings of our study have several implications for research. First, our study contributes to the literature on newcomer socialization by identifying coworker ostracism as a potential antecedent of proactive socialization. Previous studies have explored the role of supervisor support (Kammeyer-Mueller et al., 2013), team goals (Chen et al., 2008), and social networks (Morrison, 2002) in predicting proactive socialization. However, less attention has been paid on the effect of coworker ostracism. Previous research has illustrated that ostracism was detrimental to employees’ job satisfaction (Eickholt and Goodboy, 2017), in-role behaviors (Thau et al., 2015; Fatima, 2016), and justice perceptions (Verbos and Kennedy, 2015). This study extends this line of research by identifying coworker ostracism as an important antecedent for proactive socialization. Accordingly, this study sheds light on the role of coworker ostracism in newcomer socialization and reveals the negative impact of coworker ostracism during socialization process.

Second, our study contributes to ostracism literature by exploring the negative influence of coworker ostracism in the Chinese context. Confucianism defining five cardinal role relations (called *wu-lun*) is dominant in Chinese culture. Under the influence of Confucianism, individuals tend to themselves as interdependence with their surrounding (Tsui and Farh, 1997), which makes them carefully handle the relationships with colleagues. In this instance, newcomers have to endure rather than revenge when encountering coworker ostracism. Accordingly, this study enriches ostracism literature by illustrating how coworker ostracism affects newcomers’ proactive behavior during the organizational entry in the Chinese work context.

Third, we enrich the COR theory by the explication of psychological availability as a key mechanism through which coworker ostracism affects newcomers’ proactive behaviors. The literature is lacking in terms of studies on the psychological resource states of ostracism targets. Although several studies have applied resource theory to understand the consequences of ostracism (Liu et al., 2021), there is still short of research on the mediating role of psychological availability. Grounded in COR theory, our empirical research establishes a link between coworker ostracism, psychological availability, and proactive behaviors, and demonstrates that psychological availability can mediate the effect of coworker ostracism on proactive behaviors.

### Practical Implications

There are several practical implications in our study. First, this study found that coworker ostracism can impede the newcomers’ proactivity. In the organization, negative effects of coworker ostracism should not be neglected. The organization should take necessary measures to prevent the occurrence of coworker ostracism. For example, organization should devote to establish a harmonious relationship between coworkers and newcomers by creating a relaxed and pleasant working atmosphere and strengthen the construction of organizational group. In do so, newcomers can feel the care and support from the organization. In addition, organization can guide coworkers to recognize how important they are in providing feedback and support, and encourage them to play a constructive role in newcomers’ adjustment.

Moreover, given our findings that psychological availability can serve as mediator in “coworker ostracism–newcomers’ proactive behaviors” relationship, improving the psychological availability of newcomers should be paid attention. Supervisors can help newcomers decrease uncertainty and stress by clarifying their roles and expectations to improve their psychological availability (Binyamin and Carmeli, 2010). In addition, the organization should help newcomer tackle stress or negative emotion by providing psychological assistance such as emotion regulation training.

### Limitations and Future Research

Our study has several limitations. First, given that this study only proposed one possible pathway (i.e., psychological availability) to explain how coworker ostracism affected newcomers’ proactive behaviors, future research should be able to explore more possible mediators based on different theories. For example, newcomers’ attachment may also explain the mechanism between coworker ostracism and newcomers’ proactive socialization on the basic of attachment theory (Sumer and Knight, 2001).

Second, this study investigated the moderating role of emotional intelligence on the effects of coworker ostracism toward newcomers. Future research can examine whether other factors may moderate the effects of coworker ostracism on newcomers’ proactive socialization. For example, organizational culture, similarity of targets and ostracizers, and gender (Mickes et al., 2012) may be considered as moderators to further explore possible boundary conditions of coworker ostracism.

Finally, we collected data in a Chinese context. Confucianism, emphasizing mutual respect, social etiquette, and politeness, is dominant in Chinese culture. Under the influence of Confucianism, individuals tend to themselves as interdependence with their surrounding (Tsui and Farh, 1997), which makes them spend more time and energy on the relationships with colleagues. So, individuals who suffer from ostracism in a Confucian culture are more inclined to endure while those in an individualistic culture may tend to fight back (Chen and Tjosvold, 2007). Accordingly, the generalizability of our findings to other cultural contexts may be limited. Future research should collect data from newcomers in different cultural settings, to improve the generalizability of these findings.

## Data Availability Statement

The original contributions presented in the study are included in the article/supplementary material. Further inquiries can be directed to the corresponding author/s.

## Ethics Statement

This study was carried out in accordance with the recommendations of ethical guidelines of the Ethical Review Board of Beijing Jiaotong University. The protocol was approved by the Ethical Review Board of Beijing Jiaotong University. All subjects gave written informed consent in accordance with the Declaration of Helsinki.

## Author Contributions

PL made substantial contributions to the conception of the work and wrote the manuscript. YZ made contributions to the analysis of data for work. YJ contributed to the acquisition of data for work. SW revised it critically for important intellectual content.

## Conflict of Interest

YJ was employed by company CCCC Wuhan Harbour Engineering Design & Research Corporation Limited. The remaining authors declare that the research was conducted in the absence of any commercial or financial relationships that could be construed as a potential conflict of interest.
